# Empowering marginalised groups through co-operative inquiry: Illustrated by a practical example

**DOI:** 10.4102/ajod.v13i0.1205

**Published:** 2024-02-26

**Authors:** Jerome P. Fredericks, Surona Visagie, Lana van Niekerk, Hamilton G. Pharaoh

**Affiliations:** 1 Division of Occupational Therapy, Faculty of Medicine and Health Sciences, Stellenbosch University, Cape Town, South Africa; 2 Centre for Disability and Rehabilitation Studies, Faculty of Medicine and Health Sciences, Stellenbosch University, Cape Town, South Africa; 3 Division of Occupational Therapy, Department of Health and Rehabilitation Science, Faculty of Medicine and Health Sciences, Stellenbosch University, Cape Town, South Africa; 4 Communities of Excellence and Chairman Founder, Cape Town, South Africa

**Keywords:** wheelchair users, minibus taxis drivers, caregivers, access, accessibility, transport

## Abstract

**Background:**

Cooperative inquiry gives a voice to marginalised groups and breaks down power imbalances which makes it suitable for researching practical issues at community level.

**Objectives:**

The objective of this article is to illustrate how cooperative inquiry can be utilised to empower members of marginalised communities in facilitating social change.

**Method:**

The study setting is in Paarl, Western Cape, South Africa. A cooperative inquiry methodology was used. The inquiry group consisted of wheelchair users (9), their care givers (8), taxi drivers (7) and stakeholders (4). Data collection comprised 16 sessions, alternating between action and reflection. Inductive thematic analysis of data of all the phases was done to ensure that cooperative inquiry gives voice to marginalised communities.

**Results:**

The four themes that is, practical arrangements, understanding process, purpose, bonding and a cohesive group were identified. The themes showed progress from logistics, through individual understanding, to the group becoming one, and working together. Each of these phases is important in the development of a cooperative inquiry.

**Conclusion:**

Cooperative inquiry methodology can bring people together in a positive way to facilitate social change, and developing practical solutions to challenges.

**Contribution:**

Making use of a cooperative inquiry methodology to bring social change, minibus taxi services can be made accessible for wheelchair users. Concepts of social justice and decolonisation were imbued in the methodology.

## Introduction

Drawing from a study that sought to develop strategies that can assist wheelchair users to access minibus taxis, this article illustrates how cooperative inquiry can be utilised for the empowerment of members of marginalised groups. The focus of this article, however, is not to share the actual strategies that were developed. It focusses on sharing the processes followed to ensure buy-in and authentic engagement of two diverse groups who have a history of an uneasy relationship in South Africa (Kahonde, Mlenzana & Rhoda 2010; Lister & Dunpath 2016; Venter et al. 2002; Vergunst et al. 2015; Visagie, Visagie & Fredericks 2022) that is, wheelchair users and minibus taxi drivers.

The cooperative inquiry formed part of the lead researcher’s Doctor of Philosophy (PhD). It was done in a semi-rural South African town, Paarl, where minibus taxis are the main mode of public transport. The cooperative inquiry facilitated empowerment and unlocked knowledge in a marginalised, Global South community. A social justice ethos and decolonisation approach were imbued in the study’s methodology.

Research underpinned by a social justice ethos can give minority groups a voice. Eliciting these voices is important in culturally responsive research, counteracting the domination of oppressive narratives and conventional positivist research (Chilisa 2012). Marginalised groups, such as persons with disabilities, are excluded from mainstream social, economic, educational and/or cultural life. They are subjected to systematic injustice and inequity (Parrish 2018).

Members from marginalised groups are often seen as the objects of research rather than equal thinkers and knowledge bearers (Ned 2022). When researchers do not collaborate and partner with marginalised groups to create new ways of knowing, being and doing, injustice is perpetuated (Ohajunwa & Mji 2021).

Because of colonialisation, one knowledge set is considered superior while others, often indigenous knowledge sets, are ignored or ridiculed as being inferior (Ned 2022; Wooltorton et al. 2020), thereby disadvantaging and disempowering indigenous groups (Waldron 2010). Decolonisation supports the visibility of difference and diversity. It focusses on the knowledge of indigenous people as well as on their being and their doing. It normalises othered narratives and questions the dominance of a single perspective or worldview (Ohajunwa & Mji 2021).

Decolonisation is about respectful engagement with members of communities rather than the linear top-down approaches that dominated in the past (Wooltorton et al. 2020). Research with indigenous people should therefore start by acknowledging their culture and practices, and consciously affirming their knowledge and ability to solve their own problems (Ohajunwa & Mji 2021). The indigenous group in the current inquiry is the so-called ‘coloured’ people who emerged as uniquely South African with ancestors from different ethnic groups including San, Khoekhoe, Khoe-San, black African, European and Asian populations.

Cooperative inquiry inherently supports social justice and decolonisation as study participants become co-researchers and infuse research methods and findings with their value systems, worldviews and unique contextual realities. Co-researchers draw on indigenous knowledge and strengths to frame objectives, develop theoretical knowledge and/or solutions to practical problems regarding issues concerning them (Wooltorton et al. 2020). They are provided an opportunity to share their lived experiences, tell their stories and experience empowerment with the belief that their worldview is valid (Ohajunwa & Mji 2021). It is crucial that the voices and narratives of those living on the margins are heard and that they contribute to the construction of knowledge. When researchers do not collaborate with marginalised people, ill-advised and inappropriate solutions might be developed and implemented on issues relevant to them. Their involvement enhances community buy-in and thus the success and sustainability of interventions (Ohajunwa & Mji 2021).

### Cooperative inquiry

**FIGURE 1:**
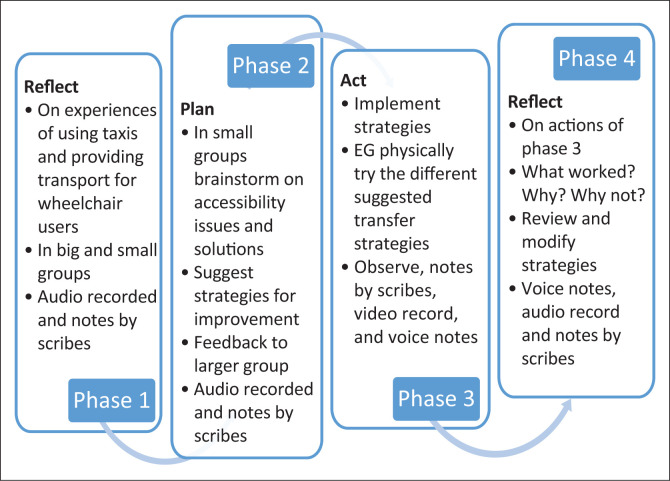
Schematic presentation of the four phases of the cooperative inquiry.

Cooperative inquiry is a form of action research that seeks meaning and develops knowledge in four distinct phases during a research cycle; it usually comprises four or more cycles (Heron 2014; Wooltorton et al. 2020). Each cycle starts with a reflective planning phase, then moves to an action phase which is followed by a reflective review, and finally further planning of the next action phase, as presented in Figure 1.

**FIGURE 2:**
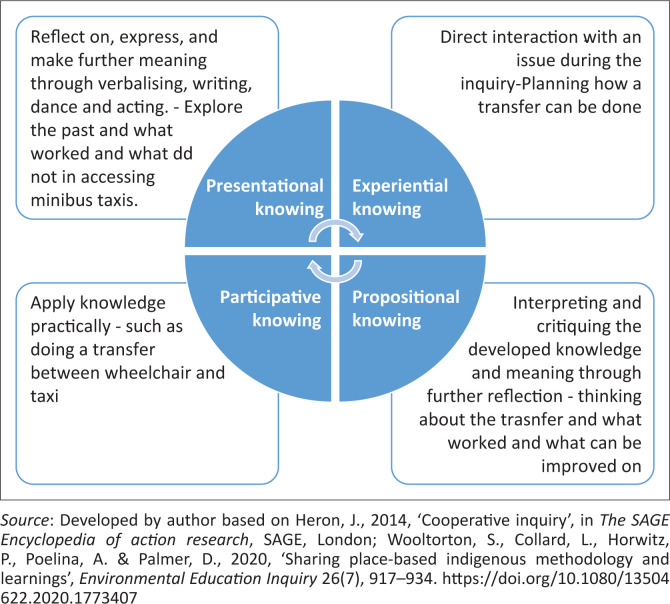
The four types of knowledge sought in cooperative inquiry.

Different forms of knowledge are built in each phase of cooperative inquiry (Figure 2). After the first cycle, knowledge is usually tentative and not well-founded. With repetition of cycles, depth of knowledge and credibility are developed. The inquiry is stopped when the group is satisfied with the outcome (Heron 2014; Wooltorton et al. 2020).

In cooperative inquiry, participants and research team members share power and take joint responsibility for the processes and decisions. All parties act as co-researchers in the planning phases and co-participants in the action phases (Heron 2014). All co-researchers and co-participants are equal.

Co-researchers must be extensively and authentically engaged, completely expressive, wholly heard and fully influential in decision-making. The process, the outcomes and the knowledge created belong to all who contributed to the inquiry (Heron 2014).

The integration of the four forms of knowing and the empowerment of participants as co-researchers make this methodology suitable for research in disadvantaged communities and/or indigenous groups. Cooperative inquiry counteracts views that might have developed over time, namely that disadvantaged groups’ knowledge is of lesser value and that their opinions *count for nothing* (Ohajunwa & Mji 2021; Wooltorton et al. 2020). Cooperative inquiry thus helps to give voice to disempowered persons and to level the power imbalance traditionally found between researchers from academia and study participants from disadvantaged groups.

Reflection, storytelling and the use of drawing can build on disadvantaged groups strengths and help them find their voices. Reflection, together with various creative techniques such as storytelling and drawing, can help identify co-researchers strengths and facilitate them finding their voices. The cyclic process and pace determined by the group help ensure all participants and/or co-researchers remain together in developing wisdom (Wooltorton et al. 2020).

Cooperative inquiry predicates that the answer to challenges can be found within communities and that all humans have useful knowledge and experiences on topics relevant to their lives. Cooperative inquiry provides a platform for exploring this knowledge and their experiences. Furthermore, it facilitates social justice as it allows participants to inform decisions about the methods, contribute to findings generated and interpret conclusions drawn in research involving them. As such they shape how the knowledge that concerns them should be formulated (Heron 2014).

A cooperative inquiry method comprising four cycles, each with four phases, is reported on in this article. The specific focus of this article is on the conditions that fostered empowerment of co-researchers, and thus allowed true participation and co-construction of knowledge. Study findings generated in the process will be presented in future articles.

### The study context

This study took place in Paarl, a peri-urban area of South Africa’s Western Cape province. Compared to adjacent communities, Paarl East is a marginalised area. Most people living in Paarl East have low education levels and experience poverty. The area was further affected by an economic downturn, job losses and a rising unemployment rate, with a resultant decrease in household income. High rates of teenage pregnancy, high school dropouts and crime have been common in the area. With rising inflation, many families were forced into poverty. Quality of life has been reduced by drug-related crimes, which has negatively impacted human development as it affects all aspects of society, such as family structures, health, the work environment and the community’s economy.

Within the Paarl community, as in other South African communities, wheelchair users live on the margins because of attitudinal barriers, stigmatisation and exclusion from services such as public transport (Lorenzo, Van Pletzen & Booyens 2015; Vergunst et al. 2017; Visagie & Swartz 2017).

Persons with disabilities are often unemployed and survive on a disability grant of ZAR2080 (±US$104) per month in South Africa (Government of South Africa 2023). The combined barriers imposed by disability and poverty reduce access to education, healthcare, employment and other socio-economic opportunities (Parrish 2018). A lack of access to transport contributes to the inability to commute in the community and makes it difficult for wheelchair users to access shops, churches and other services (Visagie et al. 2023).

In the Western Cape province, wheelchair users who do not have their own vehicle or access to a family vehicle have indicated several transportation options each with advantages and disadvantages (North & Visagie 2020; Visagie et al. 2023). None of these options were ideal. Some wheelchair users were assisted by a neighbour or a friend with a vehicle (car or pickup). They preferred this option because of the convenience of being picked up at home and dropped off at their destination. Transferring into and out of the vehicle was often easier than getting into and out of minibus taxis. However, they were dependent on the time schedule of the vehicle owner. Costs were dependent on individual vehicle owners and varied between having to contribute to fuel costs, to being charged fees up to 10 times more than the cost of a minibus taxi trip or a bus ticket (North & Visagie 2020; Visagie et al. 2023). Regarding public transport, except for a few routes in urban South Africa, train and bus services are inaccessible. Uber and other similar services have the same advantages as paying for a private car, but are too expensive for most wheelchair users (North & Visagie 2020; Visagie et al. 2023). Minibus taxis are affordable, but wheelchair users find it challenging to embark and disembark. and are often hurried by impatient drivers and fellow commuters (Fredericks & Visagie 2013; Kahonde et al. 2010; Lister & Dunpath 2016; Venter et al. 2002; Vergunst et al. 2015; Visagie et al. 2023).

Minibus taxis are one of the most important features in the South African public transport industry. Being both the most available and the most affordable form of public transport, it ferries millions of South African commuters to and from work and other essential activities. They are especially indispensable in low socio-economic peri urban areas (Fobosi 2013). Minibus taxi drivers earn between ZAR6000 (US$386.8) and ZAR7000 (US$449.6) per month. Their earnings are dependent on the number of trips and the number of passengers they transport per day. More trips and passengers mean more money in their pockets. Therefore, they have a tendency not to stop for wheelchair users and other groups who are slow to get into and out of the taxi as well as to charge for the wheelchair as it takes the space that a paying customer could have occupied.

Minibus taxi drivers have an image as aggressors and antagonists on South African roads. They are often stereotyped as aggressive, dangerous and unlawful road users (Sinclair & Imaniranzi 2015). Minibus taxi drivers are generally expected to display negative behaviours that include vulgar language, speeding, disregard for traffic lights, obstructing the flow of traffic, drifting into the safety lane, reckless driving and illegal turns, stops or parking. Members of the public associate minibus taxi drivers with taxi-related violence, which is characterised by threats, corruption, brutal attacks, illegal dealings, assaults and even assassinations (Ngubane, Mkhize & Olofinbiyi 2020). Conversely, minibus taxi drivers are confronted with rude and/or abusive passengers who put them under pressure to speed to make up time, causing conflict that can escalate to intense arguments within the taxi (Ngubane et al. 2020).

The described challenges that wheelchair users experienced to access transport coupled with the importance of community mobility provided the topic of the cooperative inquiry this article draws from. It was also clear to the lead researcher that the inquiry needed to include both wheelchair users and minibus taxi drivers as co-researchers as solutions acceptable to both groups had to be sought.

## Research methods and design

### Study design

A cooperative inquiry design was implemented to bring together two opposing groups: wheelchair users and minibus taxi drivers, to seek solutions beneficial to both. The wheelchair users were also a marginalised group. The four sessions were divided into two reflective sessions, a planning session and action sessions or practical activities. The meetings were facilitated by the lead researcher and the research assistant.

### Sampling and recruitment

Five distinct groups of co-researchers participated in the study:

- Wheelchair users who were using or wanted to start using minibus taxi services.
- Care givers of participating wheelchair users.
- Minibus taxi drivers.
- Stakeholders involved in disability matters in the study setting.
- The lead researcher and first author for this article (hereafter referred to as the lead researcher) and the research assistant.

Co-researchers had to be 18 years of age or older (the age of legal consent in South Africa). Minibus taxi drivers had to have a public transport licence with a taxi permit for transporting people. Taxi drivers who drove metered taxis were excluded from the study as the focus was on minibus taxis.

Co-researchers were recruited as follows (see Table 1 for details):

- **Nine wheelchair users** were identified through a combination of contacting disabled people’s organisations (*n* = 4), approaching known wheelchair users in the community (*n* = 3), a street-based approach (*n* = 2). A street-based approach can be used by researchers to access vulnerable groups that are hard to reach as they are not formally associated with organisations, points of service delivery, workplaces or other societies from which research participants are often sampled (Ellard-Gray et al. 2015). Therefore, where wheelchair users were seen in the community they were respectfully approached and after introductions and explanations were asked if they would be interested to participate in the research. Incorporating a street-based approach increased the likelihood of recruiting hard-to-reach participants.
- **Eight caregivers** of wheelchair users participated. The ninth wheelchair user had no carer. During recruitment, wheelchair users and carers were visited at their homes. The study was explained to them, and they were asked to attend the introductory meeting if they were interested to participate in the study. Written informed consent was obtained after the introductory meeting.
- **Seven minibus taxi drivers:** The lead researcher explained the study to the chairperson of the Paarl Taxi Association Group. He provided the endorsement of the Association, expressed his interest in the study, and provided names and contact information of 19 minibus taxi drivers who might want to participate (after obtaining their permission to share personal information). On contacting them, seven gave consent and participated in the study.
- **Four stakeholders involved in disability matters:** A professional nurse who provided orthopaedic aftercare services to the community. A disability activist who was diagnosed with polio as a child. A member of the community who is interested in disability issues. A member of the public who has technical knowledge pertaining to wheelchairs.

**TABLE 1:** Number of co-researchers per category.

Sampling	Contacted	Expressed interest to participate	Recruited
Wheelchair users	23	9	9
Carers	23	8	8
Minibus taxi drivers	19	7	7
Stakeholders	8	4	4
Lead researcher and research assistant	-	-	2

**Total**	73	28	30

### Data collection

Data were collected during the 16 cooperative inquiry sessions, organised into four cycles, between June and December 2021, as per Figure 1. The sessions lasted between 60 and 120 min each. It was not always possible for all 30 co-researchers to attend sessions. Attendance varied between 75% and 100% per session. Attendance lists were kept for compensation purposes and as part of coronavirus disease 2019 (COVID-19) protocol. The COVID-19 protocols were wearing of facial masks, taking body temperature and recorded it in a register, sanitising of hands, participants had to sign attendance list next to their contact number and physical address, maintaining social distance of 1.5-m, sanitising of all equipment such as the microphone.

The larger group of 30 co-researchers was divided into smaller groups for certain sessions, such brainstorming on strategies that might enhance wheelchair users’ access to minibus taxis and physically tying out these strategies during action phases. The smaller groups typically contained five to six co-researchers. Each small group consisted of wheelchair users, caregivers, minibus taxi drivers, and a stakeholder involved in disability matters.

Planning and reflection sessions were digitally audio-recorded, and handwritten notes were kept by four co-researchers (S2, C4, TD4 and C5 – Table 2). The practical sessions (Figure 1) were video recorded by a professional videographer supported by three co-researchers (WCU6, TD3 and TD4 – Table 2) who recorded proceedings with their cell phones.

**TABLE 2:** Co-researchers demographic details.

Participant number	Gender	Age in years	Diagnosis	Profession	Income source	Amount per month	Marital status
**Wheelchair users (WCU)**							
WCU1	Male	50	Spinal cord injury	Unemployed	Disability grant	R2080 $109.23 (USD)	Married
WCU2	Female	49	Cerebrovascular accident	Unemployed	Disability grant	R2080 $109.23 (USD)	Married
WCU3	Male	52	Cerebrovascular accident	Unemployed	Disability grant	R2080 $109.23 (USD)	Married
WCU4	Male	57	Cerebrovascular accident	Unemployed	Disability grant	R2080 $109.23 (USD)	Unmarried
WCU5	Male	55	Spinal cord injury	Unemployed	Disability grant	R2080 $109.23 (USD)	Married
WCU6	Male	32	Spinal cord injury	Unemployed	Disability grant	R2080 $109.23 (USD)	Married
WCU7	Female	54	Trans tibial amputation	Unemployed	Disability grant	R2080 $109.23 (USD)	Unmarried
WCU8	Male	67	Spinal cord injury	Unemployed	Pension grant	R2080 $109.23 (USD)	Married
WCU9	Male	37	Spinal cord injury	Employed	South African Police	R1°513°301 US$803.91	Married
**Carers (C)**							
C1	Female	63	N/A	Retired	Pension grant	R2080 $109.23 (USD)	Divorced
C2	Male	51	N/A	Employed	Tradesman	R1°090°055 US$578.82	Married
C3	Female	45	N/A	Unemployed	No income	-	Unmarried
C4	Male	28	N/A	Unemployed	No income	-	Unmarried
C5	Female	48	N/A	Unemployed	No income	-	Married
C6	Female	67	N/A	Retired	Pension grant	R2080 $109.23 (USD)	Married
C7	Female	55	N/A	Unemployed	No income	-	Married
C8	Female	41	N/A	Employed	Casual worker	Not disclosed	Married
**Minibus Taxi drivers (TD)**							
TD1	Male	44	N/A	Employed	Taxi driver	R 846°703 US$449.6	Married
TD2	Male	32	N/A	Employed	Taxi driver	R 846°703 US$449.6	Unmarried
TD3	Male	48	N/A	Employed	Taxi driver	R 846°703 US$449.6	Married
TD4	Male	45	N/A	Employed	Taxi driver	R 846°703 US$449.6	Married
TD5	Male	48	N/A	Employed	Taxi owner/driver	Not disclosed	Married
TD6	Male	38	N/A	Employed	Taxi driver	R 846°703 US$449.6	Unmarried
TD7	Male	37	N/A	Employed	Taxi driver	R 846°703 US$449.6	Unmarried
**Stakeholders involved in disability matters (S)**							
S1	Male	66	Poliomyelitis	Retired	Pension grant	R2080 $109.23 (USD)	Unmarried
S2	Female	62	N/A	Retired	Pension grant	R2080 $109.23 (USD)	Divorced
S3	Male	43	N/A	Employed	N/A	Not disclosed	Married
S4	Male	34	N/A	Employed	N/A	Not disclosed	Married
Lead researcher	Male	45	N/A	Employed	-	Not disclosed	Married
Research Assistant	Male	50	N/A	Employed	-	Not disclosed	Married

To assist co-researchers with preparation and to enhance the richness of data, a summary of previous work and points to be addressed during the next session were sent to all via WhatsApp or in hard copy (depending on their choice) before every session. The group decided when to discuss a point in more detail and when to move on to the next point. All data were transcribed and provisionally analysed after each session. Data saturation that is, the group felt that they have identified suitable strategies that might help wheelchair users access minibus taxis, was reached at the end of the third cycle. A fourth cycle was included to reflect deeper on the experiences of the co-researchers and enhance the credibility of the developed strategies.

Co-researchers communicated using WhatsApp, which is an instant messaging application. All WhatsApp messages were included as data with the permission of the co-researchers. The lead researcher kept a reflective journal, which also formed part of the data.

### Data management and analysis

Data were transcribed and analysed immediately following collection to inform subsequent phases and cycles. Video recordings added detail to transcripts, for example, to identify individual speakers, facial expressions, and body language as well as descriptions of physical activities such as boarding a taxi. Braun and Clarke’s (2012) six-step inductive thematic analysis approach was used to develop themes (Braun & Clarke 2012). An iterative reviewing and refining process was followed during which the authors reached consensus on themes. Data were collected and analysed in Afrikaans. Quotes used for reporting purposes were translated into English. The second author of this article verified the correctness of the translations from Afrikaans to English.

### Trustworthiness of the study

Trustworthiness was sought through conventional strategies used in qualitative research as well as specific strategies suggested for cooperative inquiry. Data from different sources and data collection methods were triangulated to enhance credibility, confirmability and dependability. A detailed description of the research setting and methods was done (Nowell et al., 2017) to support transferability. Repeating phases and cycles as well as movement between reflection and action phases allowed for development of deeper insights and refinement of solutions and thus enhanced credibility. Trustworthiness was also supported by the researcher’s reflection.

### Ethical considerations

Ethical approval was obtained from the Health Research Ethics Committee of Stellenbosch University (S21/01/009). Informed consent included providing information pertaining to the amount of time and level of commitment required. All co-researchers gave written consent. A first aid officer was present during the practical sessions, which included minibus taxi transfers. The first aid officer ensured safety and was on standby to provide emergency treatment should any injuries occur. No one was injured during the inquiry. Co-researchers were compensated for the time they invested by means of cash payments. The transcriber signed a declaration safeguarding personal details of the co-researchers. To support confidentiality and privacy, no video recordings were made of the planning and reflection sessions. Only the lead researcher viewed the video recordings of the practical sessions and some of the video material was shared with the supervisors. The data were stored in the password-protected Stellenbosch University’s SUN Scholar Research Repository (where they will be kept for 5 years).

## Findings

In line with the purpose of this article, the findings report on all cooperative inquiry process-related aspects from all the datasets of the 16 sessions, rather than the actual study outcomes.

### Demographic details

The demographic details of the 30 co-researchers are presented in Table 2.

### Key processes that supported the development of cooperative engagement

Four emerging themes captured the elements that were deemed critical to working together to develop cooperative solutions. These were ‘practical arrangements for a cooperative inquiry’, ‘understanding process and purpose of a cooperative inquiry’, ‘bonding of co-researchers’ and ‘formation of a cohesive group’. The categories comprising these themes are depicted in Table 3.

**TABLE 3:** Themes and categories that emerged from the data.

Practical arrangements for a cooperative inquiry	Understanding process and purpose of a cooperative inquiry	Bonding of co-researchers	Formation of a cohesive group
• Venue	• Home visits	• Building trust	• Connected
• Breaking bread	• Introductory meeting	• Coming together	• Believe
• Time	• Group contact	• Authentic collaboration	• Coming to an end
• Transport	-	• Hostility	• Moving on
• Group communication	-	-	-
• Language choice	-	-	-

#### Theme 1: Practical arrangements for a cooperative inquiry

Theme 1 demonstrated that careful consideration of practical arrangements is required because they provide the structure for the inquiry and mirror the nature of relationships and level of participation that can be expected. Co-researchers seemed to take their cue from the respectful engagement and attention to detail which was experienced during their enrolment into the study. Six categories, as shown in Table 3, captured the range of practical arrangements that fostered participation which are discussed below.

**Venue:** The size, accessibility and suitability of the venue in terms of socio-cultural-spiritual connotations required consideration. The venue had to have enough space for practical sessions and had to be wheelchair accessible. The cost of the venue and lock-down restrictions during the COVID-19 pandemic also had to be considered. A church was offered by a pastor and church council as venue. While it met the study requirements in terms of accessibility and size, sensitivity had to be shown to the fact that co-researchers, who had diverse religious affiliations, might have reservations about meeting in a church. Co-researchers were invited to share any concerns about the church venue during initial home visits as part of recruitment but in the introductory meeting none were voiced. One co-researcher who is Muslim said that:

‘I have no objections meeting at a church venue. All that I am requesting is to wear my Taqiyah [a rounded skullcap] because we as Muslims normally wear it during the day and especially when we pray.’ (TD6, 38, male)

This request was honoured.

**Breaking bread:** Eating together addressed practical, social and psychological aspects. Sessions ran over dinner time; thus, co-researchers were provided with a cooked meal at the end of each session. A community member known for making delicious food cooked the meals. The lead researcher bought the ingredients and delivered them at her house. She prepared the meals such as curry chicken, biryani, or chicken pie, salads and desserts (small, sweet treats). Cold and hot beverages were available. The food was placed in serving dishes and co-researchers dished their own food and assisted those who needed help. Money for catering was included in the cooperative inquiry budget and careful records were kept ensuring accountability. Any leftover food was placed in ‘doggy bags’ and co-researchers could take them home to share with family members. Because eating together on special occasions forms part of the culture of the community, it facilitated trust and social bonding. Being served a specially prepared meal signalled to the co-researchers that they were valued as people and for their contribution to the inquiry. Sharing a meal together allowed relationships to develop naturally and fostered a kinship between co-researchers, as WCU 9 told the group:

‘You are a wonderful group of people, the jokes we share with each other; we do not know whom among us talks most and who eats most, but I want to say to you, “You are a wonderful, wonderful group of people”. (WCU 9, 37, male)

Individual needs (such as Halal food for Muslim co-researchers) were met, and milestones (like birthdays) were celebrated during this social time.

**Time:** It was imperative that meeting times suited all co-researchers because of the goal to have all co-researchers present at every meeting. The group agreed to meet in the evening as minibus taxi drivers worked during the day. To ensure consistency, the group decided on mid-week meetings on Thursday and, if required, an additional meeting on Sundays. To avoid confusion or miscommunication, the date and time for the next meeting were set at the end of each meeting:

‘On the day of the introductory meeting, a valuable lesson was learnt regarding communicating times clearly. Around 05:15 in the morning I received a phone call from one of the co-researchers who was waiting for me to pick him up. I realised that I should have explained to him groups will be in the afternoons and not in the mornings. The effort he had to go through with his wheelchair to get to the pick-up point moved my heart because the surfaces were uneven, the area was dangerous, it was cold, he was waiting for me in the dark and I was not there to pick him up. I apologised and assured him it will never happened again. He in turn felt that it was his mistake due to his excitement to attend the groups. We laughed about the incident and put it behind us.’ (Lead researcher: Reflective journal, 45, male)

**Transport:** Access to transport was at the core of this study and, as already described, it is a complex matter for wheelchair users. As people were approached to participate in the study, their first concern was how they would get to the venue. It was important to ensure everyone could attend every meeting without anxiety about transport. To this end, the logistics included ensuring that all co-researchers had transport to all meetings and that transport costs were covered by the inquiry. Knowing that transport costs were covered and that a designated person would pick them up at a specific point meant that co-researchers could relax and focus on the inquiry.

**Group communication:** Clear and timeous communication among co-researchers was essential to the success of the inquiry. Two communication groups (one for minibus taxi drivers and one for wheelchair users and carers) were created on WhatsApp. Telephonic and in-person communication served as back up when the co-researchers did not have data to use WhatsApp. Initially, two WhatsApp groups were deemed feasible as some initial information was relevant only to the wheelchair users and other only to the minibus taxi drivers. However, only one group would have been better and assisted further in creating a unified group.

**Language choice:** The group collectively decided to use Afrikaans as medium of communication because Afrikaans was their mother tongue and the language they preferred. It was important to select a language for group communication by group consensus early in the process, as it affirmed co-researchers’ preferences, and it showed that the lead researcher was serious about the process being consultative and that the co-researchers’ opinions were being valued and acted on.

#### Theme 2: Understanding process and purpose of the cooperative inquiry

Theme 2 captured requirements that enabled study participants to perform their role as co-researchers. The theme demonstrated how home visits during participant recruitment, an introductory meeting, and development of a group contract assisted in creating the necessary understanding of processes to be followed and the purpose of the cooperative inquiry among co-researchers.

**Home visits:** Cooperative inquiry is built on relationships characterised by respect and trust. In this study, time was taken to visit potential participants in their homes in order to explain the study process and purpose, and to address their questions and concerns individually and in private. This action showed potential participants that they were respected and considered important. The potential participants were not asked to decide whether or not they would participate in the study during the visit to their homes. Instead, they were left with an invitation to attend an introductory meeting. This strategy was used to emphasise and protect their autonomy. Spending time in participants’ homes and meeting their families also helped the lead researcher to get to know the participants and to develop an understanding of their individual contexts.

The lead researcher reflected about these home visits the following way:

‘Doing home visits is one of the best ways to establish trust. You enter their [*potential participants*] homes. Being humble. Showing that you need their help and that they are important. They were provided with information about the study and could ask questions in private. Without any peer pressure. It seemed to the lead researcher that connecting with people in their homes helped them to better understand the study and their roles and they were more willing to participate in the study.’ (Lead researcher: Reflective journal, 45, male)

Home visits and spending time explaining the purpose of study clarified misunderstandings, as shown by the explanation provided by WCU 4 on his initial thoughts about the reason for the visit:

‘I thought this man is coming to win votes. Since I was busy, I quickly said I vote for no one. I am not going to vote. He then said, ‘No I am not here about voting.’ Then he explained what it was about, and I said, ‘That is something good and good to get out of the house.’ (WCU4, 57, male)

**Introductory meeting:** Individual explanations were followed by explanations and discussions in the group. Therefore, the first meeting served as a formal introduction to the study. Co-researchers had time to get to know each other, to discuss the inquiry purpose and process among themselves, and to seek further clarity from the lead researcher or the research assistant.

The study aim, the process of cooperative inquiry, and the role of co-researchers were introduced by means of a PowerPoint presentation. Following the presentation, the co-researchers divided themselves into pairs to discuss the information. They were encouraged to think of aspects they found concerning and to share their thoughts on the proposed study. Questions related to practical considerations and group logistics (e.g. number and time of meetings, compensation for time and refreshments) as well as ethical concerns and justice (e.g. ownership and use of the information, how the study will benefit co-researchers, and what will happen on completion of the study). This is reflected by the question quoted below that TD3 asked the lead researcher:

‘What is the purpose of us having these meetings and what will be done with the information provided by us.’ (TD3, 48, male)

During the introductory meeting, the co-researchers decided on practical logistics such as language use, meeting times and allocation of specific roles. They also discussed issues pertaining to the significance of the study and its possible benefits. Lastly, the co-researchers jointly developed a group contract and provided written consent.

**Group contract:** The inquiry was owned by the co-researchers who developed a contract to explicate and guide practical issues, behaviour, confidentiality and respect. The purpose of the group contract was explained as follows by the research assistant:

‘As you all know with any group engagement we need to adhere to the rules and regulations of the group and specifically when it comes to each other’s personal information. The purpose of the group contract is to talk about the do’s and the don’ts or what is acceptable or not acceptable or with what you are comfortable or not comfortable.’ (RA, 50, male)

Confidentiality, respect and equality were important to co-researchers, as illustrated by the following quotes:

‘All information should be treated as confidential and not be shared outside the group without permission.’ (WCU1, 50, male)

‘All group members must be treated with respect. Everyone must be encouraged to contribute to the discussion. All must have a fair opportunity to express their opinions. No one should laugh at another’s contribution. When one person is talking, others should listen.’ (TD2, 32, male)

‘No private side conversations while someone is speaking should be allowed and cell phones should be switched off, on silent, or vibration mode and lastly consent from the group is required to take photos and post it on social media like on WhatsApp groups.’ (S2, 62, female)

Important elements were reinforced by the research assistant as shown in the example below:

‘Inappropriate language like swearing or making inappropriate comments to each other or the opposite sex are not permissible.’ (RA, 50, male)

#### Theme 3: Bonding of co-researchers

Co-researchers ascribed the success of the inquiry reported here to the development of an emotional bond among co-researchers. This required the building of mutual trust, coming together in the group, authentic collaboration and dealing with hostility.

**Building trust:** Developing mutual trust was especially important in this study because of existing power imbalances between wheelchair users and minibus taxi drivers, as well as between wheelchair users and minibus taxi drivers on one hand and the lead researcher and research assistant on the other. Some co-researchers shared doubts about their participation in the study:

‘I am sceptic to participate because there is no political vote in this country for wheelchair users. Nothing! Nothing! All the ministers at the President’s office knows about all of our challenges but still they are doing nothing to assist us. They don’t want to do anything for us. So what is the use to participate and you know nothing, nothing is about to happen.’ (S1, 66, male)

They feared disappointment and being misused:

‘In the past we had so many meetings and so many promises have been made and still today nothing has changed so what will be the difference with these types of meetings?.’ (S1, 66, male)

The research assistant acknowledged the co-researchers’ fears and helped create a safe and trusting space where knowledge could be shared and developed. The research assistant shared that:

‘We live in a community where everybody is always in crises. Our community is continually in trauma, our people experience trauma, but there is no one to assist them through the trauma. Every one of us walk with pain every day. We need deliverance and we need to be there for one another and support each other. Tonight, we can make the choice about all our pain and decide we want to make something beautiful from it. I think that is what the future holds for us, whatever develops from this [*the inquiry*], the next time when it is not inquiry, can we get together and say, “What can we build from here?” That is part of the work that Jerome and I want to do … that is why we are together, it is not about inquiry, not about a doctoral degree. It is about how can we make a difference in our community to give more hope to someone else.’ (RA, 50, male)

The lead researcher shared his vision for the study:

‘Think of those who don’t have transport to go and see their loved one participating in sport events or receiving academic awards at school, who is unable to make use of services within their communities or who is unable to participate in leisure activities. My vison is that we as co-researchers can ultimately contribute to improved quality of life for wheelchair users.’ (Lead researcher, 45, male)

The lead researcher also shared his emotions and perspective on the importance of the study while affirming the essential contribution of co-researchers without whom the inquiry could not be done. Having recently lost his mother, who required a wheelchair in later life, he made the link between his personal aspirations and the rationale for the inquiry:

‘Looking at the wheelchair, I see my mom in it and that motivates me to continue with the study … every time I look at that chair, I experience inner strength. That chair of hers is like her saying to me, “Here is the empty chair, go and fill it, finish your PhD”.’ (Lead researcher, 45, male)

**Coming together:** For successful cooperative inquiry, people must be together in mind and spirit, not only physically. Co-researchers believed in the importance of the study and that helped to unite the group. A key moment, when the group started to discuss the need for the study, illustrated the importance of letting co-researchers express their thoughts and feelings instead of providing them with answers; co-researchers started power-sharing and a cooperative process. Wheelchair users and caregivers united in their realisation that participation in the inquiry could be the platform they had been looking for to raise their voices regarding inaccessible minibus taxi services:

‘… taxi drivers and wheelchair users don’t have an understanding with each other when making use of taxi services … this platform will assist them to raise their voices to have a better understanding for each other as well as regards to accessible minibus taxi services.’ (S1, 66, male)

The feeling, that someone was really listening to their challenges, seemed to bolster and motivate them. The group became a source of information and co-researchers grew together. As shown by the statements of appreciation made by C7 and TD6 to the group:

‘Uncle and I stayed with the group. We came to love the process we went through. This is a lovely group of people. We communicate, we learn that we must respect people in wheelchairs. I have been educated in that. I am glad I have met Jerome and that I could attend the sessions.’ (C7, 55, female)

‘I had a sense of belonging because no one judged each other. All just want to make a difference or adding value for a better circumstance for themselves as wheelchair users.’ (TD6, 38, male)

**Authentic collaboration:** It was important that everybody felt welcome to participate and know that their opinions were valued and important. The foundation for authentic participation was created through group consensus on practical issues such as the timing, duration and frequency of sessions. Co-researchers also steered the development of the group contract and picked roles that suited them. Making these decisions helped to develop a sense of autonomy and ownership among all.

The discussions were not dominated by one or two co-researchers. Everyone had the opportunity to raise their voices and share their thoughts and experiences. No voices were silenced, and all contributions were appreciated as worthy. The lead researcher explicitly reinforced co-researchers to contribute to the cooperative inquiry through statements like:

‘There is no right or wrong answer; just say it just as it is.’ (Lead researcher, 45, male)

Affirmative responses were used to facilitate participation:

‘Thank you very much for sharing that information with us. It is valuable and insightful.’ (Lead researcher, 45, male)

Not all are equally comfortable to speak in a large group. To facilitate authentic collaboration, smaller group (five co-researchers) discussions were included in the process. The research assistant also encouraged co-researchers to commit themselves to the study, cooperate and speak out:

‘We can make a difference with the study and in our community so that another person and specifically wheelchair users can have hope in the future.’ (RA, 50, male)

**Dealing with hostility:** Wheelchair users and minibus taxi drivers have generally been considered antagonists, with little cooperation and regular hostility between them (Kahonde et al. 2010; Lister & Dhunpath 2016; Lorenzo 2008; Venter et al. 2002; Vergunst et al. 2015; Visagie et al. 2023). However, in the current study, it was essential that both parties were present and working together towards a common goal. At the start of the inquiry, the relationship between the two groups was strained. The wheelchair users and caregivers made it clear that they were not happy with the treatment they received from minibus taxi drivers in the past and that it affected them in a negative way. They generalised experiences of being treated poorly by minibus taxi drivers to all the minibus taxi drivers, including those participating in the inquiry.

In one incident, a caregiver expressed emotional distress because of poor services and unfair treatment of her loved ones. She strongly expressed her dissatisfaction and disappointment towards a specific minibus taxi driver:

‘Taxi drivers don’t feel anything for wheelchair users they are all the same just to make money and they don’t care for wheelchair users. I want to say to the taxi driver most of you taxi drivers are the same. You do not care about people that use wheelchairs. Both my mother and father use wheelchairs and we cannot depend on taxis for transport. I know it will not happen. You do not have any feelings towards us; and nobody will be able to convince me differently … We know you must make money … but you will lose nothing by being a little friendlier and more helpful.’ (C8, 41, female)

The atmosphere in the group was very tense, but all co-researchers empathised with her pain. The group encouraged her to express her emotions and confirmed that she had the right to her feelings. After being gently calmed down, she felt relieved and at ease with her emotions and realised she never had the opportunity to express the grief, which she carried with her all these years. The co-researchers were validated for the decision to spend time and provide the space the caregiver needed to identify and deal with her emotional disturbance.

The minibus taxi driver was also provided with the opportunity to share his views and thoughts. He made it clear that it was not his intention to hurt the emotions of anyone and that the group assisted him to understand the challenges wheelchair users face:

‘I am very sorry if I hurt anybody’s feelings. It was not my intention. We, taxi drivers, including myself, needs training so that we can be friendlier and provide more help to wheelchair users. Drivers must go for training. And a hundred percent their mindsets must change. How you approach your passenger. I think the wheelchair thing shocked me to reality tonight. Never before, have I talked with people in a group. I now realise how difficult it is. Personally, I am going to try to assist the guy in the wheelchair to get around.’ (TD5, 48, male)

In general, the minibus taxi drivers acknowledged the frustrations of wheelchair users and pointed out that they were unaware of the struggles and how difficult it was for wheelchair users to use minibus taxis. It was also pointed out that the attitudes of wheelchair users can determine the support they get from minibus taxi drivers:

‘As taxi drivers we must realise we are here to provide a service. But if the attitude of wheelchair users is arrogant it will determine whether we will assist them and how we will act towards the wheelchair user. I can say honestly no taxi driver will assist an arrogant wheelchair user.’ (TD3, 48, male)

The conversation helped both groups to develop a better understanding of the other’s situation. This sharing brought the realisation that they needed to join hands and work together in harmony to address the challenges with appropriate and suitable strategies:

‘It is very important that we as wheelchair users and taxi drivers respect and appreciate each other and that we have patience with each other.’ (WCU4, 57, male)

‘There needs to be training for us as taxi drivers so that we can have a better understanding of the needs of wheelchair users and how we as taxi drivers can handle them and provide a better taxi service.’ (TD5, 48, male)

#### Theme 4: Forming of a cohesive group

After the forming, storming and norming stages captured in the previous themes, the final theme comprised the performing stage of the cooperative inquiry and adjourning the process. This theme consists of four categories which are: connectedness, believing, coming-to-an-end and moving on.

**Connectedness:** The connectedness among the co-researchers became stronger towards the middle of the study and a sense of community developed:

‘The groups are very important to me. The first time when I walked in, I felt the atmosphere, it is my family. Not in flesh, but spiritual. When people ask me about the group … I respond, “Those people are like my own family, I feel at home with them like with the family in my house”.’ (WCU3, 52, male)

‘In the beginning when [lead researcher] approached me to be part of this group, I did not want to come at first. But I am not sorry that I am here. I learnt a lot. Things I did not know, and it was informative, the friends, the small group that I was a part of was lovely.’ (C6, 67, female)

‘I have a sense of belonging because no one judged each other. All just want to make a difference or adding value for a better circumstance for themselves as wheelchair users.’ (TD6, 38, male)

Their engagement moved beyond the study requirements co-researchers’ roles and responsibilities with regards to the cooperative inquiry, as social relationships were developed. Co-researchers communicated electronically, visited each other, showed concern for each other’s well-being, and provided support to each other.

Wheelchair user number 6 sourced funding and organised a *Valentine’s Ball* for the co-researchers, their partners and other wheelchair users in the community:

‘I am so empowered with what we have done and achieved with the inquiry that it inspired me to do more. I would like to know if I can contact members who are part of the inquiry to ask them if they are interested to attend a valentine ball for wheelchair users free of charge.’ (WCU6, 32, male)

**Believe:** Being from marginalised communities, co-researchers might have felt they have little to offer. However, through the inquiry process they developed confidence in their ability to find solutions to problems faced; something they took pride in. For instance, WCU7 expressed that:

‘I am happy and believe God will provide a way. In the future wheelchair users will have better way to ride about in taxis.’ (WCU7, 54, female)

The lead researcher, on the other hand, shared that:

‘Every time I come to a group meeting and stand in front of you … and talk with you, I ask myself, “Goodness, we are all from [names of three suburbs] can anything good come from [first suburb], can anything good come from [second suburb], can anything good come from [third suburb]? These are usually the neighbourhoods that nobody wants to be associated with” Then I look at this group and I can say proudly, “Yes, good things can come out of these neighbourhoods”.’ (Lead researcher, 45 male)

**Coming-to-an-end:** Terminating the inquiry process came with many emotions because co-researchers valued attending weekly group discussions, experienced a growth in knowledge and confidence, developed trust, and started caring for each other as shown by statements from WCU7, C5, TD6 and C3:

‘I feel unhappy, unsure, disappointed and angry because it is almost a piece of me is taken away.’ (WCU7, 54, female)

‘I am a bit emotional now that we are getting to the end of our meetings because I have learned so much about assisting wheelchair users and people with disabilities. Yes, it is sad because I enjoy the discussions a lot.’ (C5, 48, female)

‘I will be missing the group sessions and interaction with the everyone. I met wheelchair users with disabilities who want to make a positive impact in life. I can learn from each one and was looking forward to seeing everyone at the group sessions.’ (TD6, 38, male)

‘I am happy to have been part of the group. I met new people whom I did not know before. I have learned so much … Before I came to the group, I was shy. I could not talk in front of people. The group changed me a lot.’ (C3, 45, female)

The co-researchers were reminded that they could visit a counsellor of their choice and that the cost of counselling would be covered by the current inquiry project budget.

**Moving on:** The cooperative inquiry process has equipped co-researchers with the belief that they can make a difference going forward:

‘I want to take what I have learned and not keep it for myself. I want to go out and talk about what I have received here. I want to share it with people.’ (C3, 45, female)

Some of the co-researchers started to envisage broader opportunities for persons with disabilities, for example, a driving school specifically for persons with disabilities and persons with disabilities obtaining ownership of a taxi service. Wheelchair user number 6 shared that:

‘I do not know how realistic this sounds, but I am thinking of starting my own driving school for people with disabilities and specifically wheelchair users who have the need to drive their cars again. I know a lot of planning and logistics will need to go into this idea of mine, but I would like to explore this area.’ (WCU6, 32, male)

Wheelchair user number 1 similarly expressed:

‘What if one of the wheelchair users get hold of his own taxi for wheelchair users in the Paarl area?’ (WCU1, 50, male)

Participation in the inquiry had positive spin-offs with potential community building initiatives beyond the inquiry, as captured by S2:

‘We learned so much from each other and about our needs. No one should be underestimated despite your age, culture, race, status in life or how many degrees you have. We can learn from each other. So, coming to an end it is just amazing to be part of this process and that this process should not be temporary because there are so many other aspects that still can be addressed.’ (S2, 51, male)

The minibus taxi drivers who participated in the cooperative inquiry developed an understanding of the challenges faced by wheelchair users when using minibus taxis. They expressed how the inquiry has changed their perceptions towards wheelchair users and that they would gladly assist wheelchair users with transport in future. As indicated by TD4:

‘Seeing that I have not yet transported wheelchairs users, but I have already gained experience here in the few sessions we had, it will be a pleasure for me to transport someone in a wheelchair. I have learned a lot here and I have motivated myself from the beginning until now and I would prefer to do it with communication and love for the people. I now understand the dilemma of the people with wheelchairs and for me it will not be difficult at all, on the contrary, it will be a pleasure for me to be able to transport people with wheelchairs.’ (TD4, 45, male)

Minibus taxi drivers have kept this promise and sent video clips and photos of how they are transporting wheelchair users after attending the cooperative inquiry to the lead researcher. The minibus taxi drivers used the transfer strategies developed during the inquiry to assist wheelchair users into and out of the minibus taxi.

Co-researchers felt similar inquiries can be used in future in Paarl to address other disability matters for instance facilitating the incorporation of universal design principles of buildings. Wheelchair user number 3 stated:

‘From my side after we have covered this accessible minibus taxi for wheelchair users, and we are now able to get to the shops, but the problem is look at centre point shop for instance it is not wheelchair accessible. I think the group should look at what can be done to make all buildings accessible for wheelchair users as well.’ (WCU3, 52, male)

The lead researcher had the opportunity to share about the cooperative inquiry at public events, churches, on local radio stations and on national television.

## Discussion

The themes captured the progression of the inquiry from practical arrangements for a cooperative inquiry to individual understanding of the process and purpose of a cooperative inquiry, the bonding of co-researchers to the formation of cohesive group, and finally becoming one, and working together, and moving on. Each of these phases is important in the development of a cooperative inquiry.

### Practical arrangements for a cooperative inquiry

Paying attention to, and deliberating on, practical and logistical aspects are essential components of a successful cooperative inquiry. Ensuring that practical arrangements were well organised, inclusive and welcoming set the platform for the inquiry. Giving attention to the practical arrangements is important for the success of the inquiry. It affirms co researchers and shows respect for them, their time and contributions. It provides co-researchers with a sense of organisation and peace of mind. Following a clear structure for logistical arrangements meant everybody knew exactly how the process would unfold and left little room for confusion and misunderstandings. With these basics in place, the co-researchers could focus their energy on the inquiry rather than logistical issues like transport, meeting times or what they would eat when they got home after the sessions.

It is part of South African culture to provide food during engagements. Eating together is associated with laughter and happiness; it provides an opportunity for people to interact in a relaxed manner, share jokes and enjoy one another’s company. Sharing a meal means that stressors are put aside for the moment and the opportunity is created for building trust and relationships are built. According to Dunbar (2017), people tend to feel closer to one another when eating together. Furthermore, it widens an individual’s social networks and the possibility for social and emotional support. In this study, eating together created a safe space for co-researchers to share their deeper feelings of concerns and worries with other co-researchers around the table.

Language choice is important in South Africa with its 12 official languages and the potentially divisive forces when people do not understand each other or are forced to speak a language which they are not comfortable with. Forcing a language on the group could have made some members feel unwelcome, thus stifling their opinions. Conversely, speaking in your home language facilitates spontaneity as you can express yourself without fear of using incorrect grammar or difficulty finding the exact word to clarify your thoughts. People tend to feel shy and limit their engagement in a language they are less comfortable with, because they might be worried about embarrassing themselves (Tanveer 2007). Letting co-researchers express themselves in a language that they were comfortable with, Afrikaans in this instance, allowed them to better share their views and experiences, an aspect also reported by Williams (2019).

### Understanding processes and purpose of cooperative inquiry

For members of the inquiry group to participate actively as co-researchers, they had to have a clear understanding of the purpose of the study and their roles in it. The level of involvement expected from co-researchers might have been a new experience to some co-researchers. Therefore, time had to be spent in the initial stages of the inquiry to ensure that all co-researchers understood the study processes and their possible contribution to it.

The nature of cooperative inquiry, with its phases and cycles, can be confusing. To prevent confusion, the workings of each phase and cycle had to be explained repeatedly. Facilitating understanding of the processes and purpose of the inquiry started with home visits, was reinforced at an introductory meeting, and further supported by information as the inquiry unfolded. It was important to focus on only one phase per session. Providing co-researchers with session topics in advance allowed them to come prepared and contribute meaningfully to the discussions. Furthermore, they understood what was being done in each phase and could organise their thoughts and responses.

Community entry and researchers building relationships with community members in general, and study participants specifically has been shown to be critical aspects of any research process (Chilisa 2012). Visiting potential participants in their homes affirmed the co-researchers’ worth and helped the lead researcher to understand them better through observing them in context; this finding was previously described by Kawulich (2005).

For the co-researchers in the current research, concrete activities such as breaking into small groups, discussing what they have heard and bringing that back to the larger group enhanced understanding of the methods and expectations.

### Bonding of co-researchers

It is important that intersubjective dialogue should manifest through authentic collaboration since it is a key component in refining knowing in cooperative inquiry (Heron 2014). For cooperative inquiry to succeed, co-researchers must collaborate. In this study, collaboration was premised on development of genuine empathy with each other. Empathy requires that people make themselves vulnerable and share traumatic lived experiences relevant to the topic (Day, Lawson & Burge 2017). People can only share when there is trust in the group and when they feel the space is safe (Kolbe et al. 2020). In their vulnerability, the strength and resilience of co-researchers could be seen regarding the challenges around the topic.

Hesitance and distrust, as was shown initially by co-researchers, was not surprising as researchers had in the past abused the trust of marginalised groups through disempowering research practices and the misrepresentation of their stories (Ohajunwa & Mji 2021). It is not known whether current study co-researchers had past experiences of such practices, and they might have been exposed to or heard of these practices.

When participants take part in research, an expectation is being created by the researchers that the situation will change, but sadly in some cases their situation remains the same (Cook & Cox 2022). Mc Donald (2020) warned that the academic members of a cooperative inquiry group must remain aware of the power imbalances and the undercurrents of mistrust and abuse. They should never force their opinions on the group. Their engagement with participants is not to dominate but to seek collaboration and learn (Ohajunwa & Mji 2021). It is important to ensure an equilibrium in power between the co-researchers (McDonald 2020). Through being honest with participants and sharing emotions and dreams for the study, the lead researcher showed his vulnerability. This could have assisted in breaking down distrust and helped facilitate authentic participation on the part of the co-researchers. People want to work with real people with integrity and morals who can provide hope to inspire, to empower but most of all who are making a positive change within the community (Page & Wong 2000).

In this study, there was a second power dynamic to be managed; that between wheelchair users and minibus taxi drivers. For these two parties to develop cohesion, dialogue was required at a level of sharing emotion. Both parties’ views had to be heard and the group had to collectively solve issues in order to work towards a common goal. The inquiry process lends itself towards opportunities for people to unpack and express past hurts; and then move forward collectively. Past hurt and frustration can be projected onto other members of the group as was shown in the current study findings. The natural response might be to defend oneself; a situation that might harm or even derail the inquiry (Heron 2014). In this study, it was deemed necessary to allow space for sharing pain, but then also to give everybody an opportunity to share their feelings, clear the air, and move forward as a collective. The caregiver could openly accuse minibus taxi drivers of being uncaring when it comes to wheelchair users, and the minibus taxi driver had the opportunity to respond. The group supported both of them and they could resolve the animosity and move forward collectively. Every co-researcher had to feel valued, and co-researchers trusted each other with personal and, sometimes painful, information. For optimal results, the group had to work together as a cohesive unit instead of against each other or in smaller subunits that do not trust each other. In the current inquiry, small groups were used, but these were never in opposition to each other. The smaller groups functioned as subsets of the larger group who always had the success of the larger group at heart. A sense of belonging and pride to be involved in something important such as access to minibus taxis was fostered.

When reflecting on past experiences, there is a tendency to disclose things which were avoided in the past which can produce strong emotions (Heron 2014). The findings presented under dealing with hostility demonstrated how the co-researchers’ willingness to provide space for and pay attention to the distress shown by others led to authentic sharing and fostered true participation. If distress and strong emotions were ignored, the suggestions of this inquiry, as described in another article, could have been distorted by the buried emotions (Heron 2014).

### Formation of a cohesive group

The co-researchers have never before had a platform or opportunity to share their experiences and be part of a formal process for developing solutions to a common problem. Coming-to-an-end for co-researchers came with many emotions because they felt that they will be missing out on the social interaction they had together, which included group discussions, eating together, supporting each other, learning from each other and making a positive contribution to an inquiry. Discussing the actual contribution made in this inquiry falls outside the scope of this article as the focus here is on the cooperative inquiry process. Suffice to say that co-researchers developed strategies that can be further explored and hopefully implemented to assist wheelchair users to access minibus taxis in the Paarl community, and to guide minibus taxi drivers in accepting wheelchair users as members of their consumer body. These strategies will be presented in detail in another article.

The cooked meals members received allowed some of the members to share it with the rest of their families at home. One should also take note of how the compensation has assisted the co-researchers to buy some of their basic needs for themselves and their households. Coming-to-an-end and losing out on what one could call benefits to members can be seen as lost opportunities or resources. Part of the lost opportunity was that members will not come together in the future to meet, to support, and learn from each other.

Lastly, cooperative inquiry provided a platform where members felt valued, a platform where growth has taken place, and they were contributing in a positive manner to the inquiry. While co-researchers were sad about the ending of the process, some of them were empowered to take on new challenges in the future as described under ‘moving on’. The practical activities or the active phases were the most effective because they provided the co-researchers the opportunity to apply and implement the strategies they have reflected on and planned. For instance, manual transfers were planned and then in the action phase a minibus taxi was available, and the minibus taxi drivers had to transfer a wheelchair user into and out of the minibus taxi based on what was decided on by the group. Furthermore, the co-researchers observed and made notes of what worked and what did not work. They looked at facial expressions and non-verbal communication when the wheelchair users were transferred into and out of the minibus taxi and shared their observations with each other. This led to further suggestions to improve the transfer technique. Regarding the aim of the inquiry, outcomes included communication strategies, different cost and payment options, awareness-raising, home pick up and drop off, a few minibus taxis with different access features such as ramps or hoists and docking stations, transfer techniques, and suggestions for wheelchair storage during transit. These will be described in detail in future articles.

### Strengths and limitations

Making use of a research assistant that was known to the community and skilled in qualitative research was one of the strengths of this study, as also described by Flenady et al. (2022). The research assistant’s knowledge of the context and research methods meant he could support co-researchers in an appropriate manner. Conducting home visits as part of the recruitment process created a firm foundation for building rapport, which fostered authentic participation on behalf of co-researchers.

Having two WhatsApp communication groups instead of one might have been a limitation as greater group cohesion might have been achieved with all participants sharing the same group.

## Conclusion and recommendations

Cooperative inquiry methodology can bring people together in a positive way to facilitate social change. The ability of cooperative inquiry to give voice to marginalised groups and break down power imbalances, as shown in this inquiry where wheelchair users and minibus taxi drivers became co-researchers, makes it suitable for researching practical issues, such as access to minibus taxis for wheelchair users, at community level. It provides an opportunity for people with differing viewpoints to collaborate in a non-threatening space. Co-researchers can develop an understanding of one another’s perspective and jointly create practical solutions that are agreeable to all. True collaboration, knowledge sharing and co-construction of knowledge require explicit facilitation throughout the inquiry. It is key to develop the belief among co-researchers that everyone has meaningful contributions to make. Each member in the group should have confidence in their own opinion and be spontaneous in sharing their views. At the same time, everyone should be open to consider differing opinions. Cooperative inquiry is recommended as a strategy that can be used in research and projects to assist marginalised groups to develop practical solutions to the challenges they face.

## References

[CIT0001] Braun, V. & Clarke, V., 2012, ‘Thematic analysis’, in H. Cooper, P.M. Camic, D.L. Long, A.T. Panter, D. Rindskopf & K.J. Sher (eds.), *APA handbook of research methods in psychology, vol. 2. Research designs: Quantitative, qualitative, neuropsychological, and biological*, pp. 57–71, American Psychological Association, Washington, DC.

[CIT0002] Chilisa, B., 2012, *Indigenous research methodologies*, Sage, Thousand Oaks, CA.

[CIT0003] Cook, D.J. & Cox, P., 2022, ‘A case for complexity-informed participatory action research with young people’, *Education, Citizenship and Social Justice* 17(2), 188–202. 10.1177/1746197921995153

[CIT0004] Day, K.W., Lawson, G. & Burge, P., 2017, ‘Clinicians’ experiences of shared trauma after the shootings at Virginia Tech’, *Journal of Counseling & Development* 95(3), 269–278. 10.1002/jcad.12141

[CIT0005] Dunbar, R.I.M., 2017, ‘Breaking bread: The functions of social eating’, *Adaptive Human Behavior and Physiology* 3, 198–211. 10.1007/s40750-017-0061-4 32025474 PMC6979515

[CIT0006] Ellard-Gray, A., Jeffrey, N.K., Choubak, M. & Crann, S.E., 2015, ‘Finding the hidden participant: Solutions for recruiting hidden, hard-to-reach, and vulnerable populations’, *International Journal of Qualitative Methods* 14(5), 1609406915621420. 10.1177/1609406915621420

[CIT0007] Flenady, T., Dwyer, T., Kahl, J., Sobolewska, A., Reid-Searl, K. & Signal, T., 2022, ‘Research capacity building for clinicians: How does the research facilitator role foster engagement in the research process?’, *Research Square* 1. 10.21203/rs.3.rs-1216750/v1 PMC9044663 35477479

[CIT0008] Fobosi, S., 2013, *The minibus taxi industry in South Africa. A servant for the urban poor?*, viewed 19 August 2023, from https://www.polity.org.za/article/the-minibus-taxi-industry-in-south-africa-a-servant-for-the-urban-poor-2013-05-06.

[CIT0009] Fredericks, J.P. & Visagie, S., 2013, ‘The rehabilitation programme and functional outcomes of persons with lower limb amputations at a primary level rehabilitation centre’, *South African Journal of Occupational Therapy* 43(3), 18–28.

[CIT0010] Government of South Africa, 2023, *Disability grant*, viewed 19 August 2023, from https://www.gov.za/services/social-benefits/disability-grant.

[CIT0011] Heron, J., 2014, ‘Co-operative inquiry’, in D. Coghlan & M. Brydon-Miller (eds.), *The SAGE encyclopedia of action research*, pp. 328–331, SAGE Publication, London.

[CIT0012] Kahonde, C.K., Mlenzana, N. & Rhoda, A., 2010, ‘Persons with physical disabilities’ experiences of rehabilitation services at Community Health Centres in Cape Town’, *South African Journal of Physiotherapy* 66(3), 2–7. 10.4102/sajp.v66i3.67

[CIT0013] Kawulich, B., 2005, ‘Participant observation as a data collection method’, *Forum: Qualitative Social Research* 6(2), 43. 10.17169/fqs-6.2.466

[CIT0014] Kolbe, M., Eppich, W., Rudolph, J., Meguerdichian, M., Catena, H., Cripps, A. et al., 2020, ‘Managing psychological safety in debriefings: A dynamic balancing act’, *BMJ Simulation & Technology Enhanced Learning* 6(3), 164–171. 10.1136/bmjstel-2019-000470 PMC8936758 35518370

[CIT0015] Lister, H.E. & Dhunpath, R., 2016, ‘The taxi industry and transportation for people with disabilities: Implications for universal access in a metropolitan municipality’, *Transformation: Critical Perspectives on Southern Africa* 90(1), 28–48. 10.1353/trn.2016.0009

[CIT0016] Lorenzo, T., Van Pletzen, E. & Booyens, M., 2015, ‘Determining the competences of community-based workers for disability-inclusive development in rural areas of South Africa, Botswana and Malawi’, *Rural Remote Health* 15(2), 2919. 10.22605/RRH2919 26048267

[CIT0017] Ned, L.Y., Dube, K. & Swartz, L., 2022, ‘Challenges and opportunities of centring the African voice in disability research’, *African Journal of Disability (Online)* 11, 1–4. 10.4102/ajod.v11i0.1089 PMC9634708 36338868

[CIT0018] Ngubane, L., Mkhize, S. & Olofinbiyi, S.A., 2020, ‘Taxi violence in South Africa: Insight from Mpumalanga Township, Kwazulu-Natal Province, South Africa’, *African Journal of Peace and Conflict Studies* 9(3), 81. 10.31920/2634-3665/2020/v9n3a5

[CIT0019] North, G. & Visagie, S., 2020, ‘Keeping advanced seating services appointments in a Western Cape setting: A qualitative exploration of the experiences of carers of persons with cerebral palsy’, *South African Journal of Occupational Therapy* 50(3), 64–71. 10.17159/2310-3833/2020/vol50no3a8

[CIT0020] Nowell, L.S., Norris, J.M., White, D.E. & Moules, N.J., 2017, ‘Thematic analysis: Striving to meet the trustworthiness criteria’, *International Journal of Qualitative Methods* 16(1), 1609406917733847. 10.1177/1609406917733847

[CIT0021] Ohajunwa, C. & Mji, G., 2021, ‘Expressing social justice within indigenous research: A reflection on process and affirmation’, *AlterNative: An International Journal of Indigenous Peoples* 17(2), 183–190. 10.1177/11771801211001567

[CIT0022] Page, D. & Wong, P.T.P., 2000, ‘A conceptual framework for measuring servant leadership’, in S. Adjibolooso (ed.), *The human factor in shaping the course of history and development*, pp. 1–28, American University Press, Lanham, MD.

[CIT0023] Parrish, D., 2018, ‘Evidence-based practice: A common definition matters’, *Journal of Social Work Education* 54, 407–411. 10.1080/10437797.2018.1498691

[CIT0024] Schwartz, A.E., Kramer, J.M., Cohn, E.S. & McDonald, K.E., 2020, ‘“That felt like real engagement”: Fostering and maintaining inclusive research collaborations with individuals with intellectual disability’, *Qualitative Health Research* 30(2), 236–249. 10.1177/1049732319869620 31466513

[CIT0025] Sinclair, M. & Imaniranzi, E., 2015, *Aggressive driving behaviour: The case of minibus taxi drivers in Cape Town, South Africa*, viewed 12 August 2023, from http://hdl.handle.net/2263/57797.

[CIT0026] Tanveer, M., 2007, ‘Investigation of the factors that cause language anxiety for ESL/EFL learners in learning speaking skills and the influence it casts on communication in the target language’, Masters’ thesis, University of Glasgow, Glasgow.

[CIT0027] Venter, C.J., Bogopane, H.I., Rickert, T.E., Camba, J., Venkatesh, A., Mulikita, N. et al., 2002, *Improving accessibility for people with disabilities in urban areas*, viewed 13 November 2023, from https://www.gov.uk/research-for-development.

[CIT0028] Vergunst, R., Swartz, L., Hem, K.G., Eide, A.H., Mannan, H., MacLachlan, M. et al., 2017, ‘Access to health care for persons with disabilities in rural South Africa’, *BMC Health Services and Research* 17(1), 741. 10.1186/s12913-017-2674-5 PMC5693516 29149852

[CIT0029] Visagie, W.B., Visagie, S.J. & Fredericks, J.P., 2023, ‘Community mobility: Psychosocial experiences of stroke survivors who use wheelchairs in Worcester, South Africa’, *South African Journal of Occupational Therapy* 53(1), 81–91. 10.17159/2310-3833/2023/vol53n1a9

[CIT0030] Visagie, S. & Swartz, L., 2017, ‘“There is nothing wrong with me”: Disability invisibility in a rural South African town’, *Disability and Rehabilitation* 40, 1–12. 10.1080/09638288.2017.1313909 28399720

[CIT0031] Waldron, N.L. & McLeskey, J., 2010, ‘Establishing a collaborative school culture through comprehensive school reform’, *Journal of Educational and Psychological Consultation* 20(1), 58–74. 10.1080/10474410903535364

[CIT0032] Williams, D.R., Lawrence, J.A., Davis, B.A. & Vu, C., 2019, ‘Understanding how discrimination can affect health’, *Health Services Research* 54, 1374–1388. 10.1111/1475-6773.13222 31663121 PMC6864381

[CIT0033] Wooltorton, S., Collard, L., Horwitz, P., Poelina, A. & Palmer, D., 2020, ‘Sharing a place-based indigenous methodology and learnings’, *Environmental Education Research* 26(7), 917–934. 10.1080/13504622.2020.1773407

